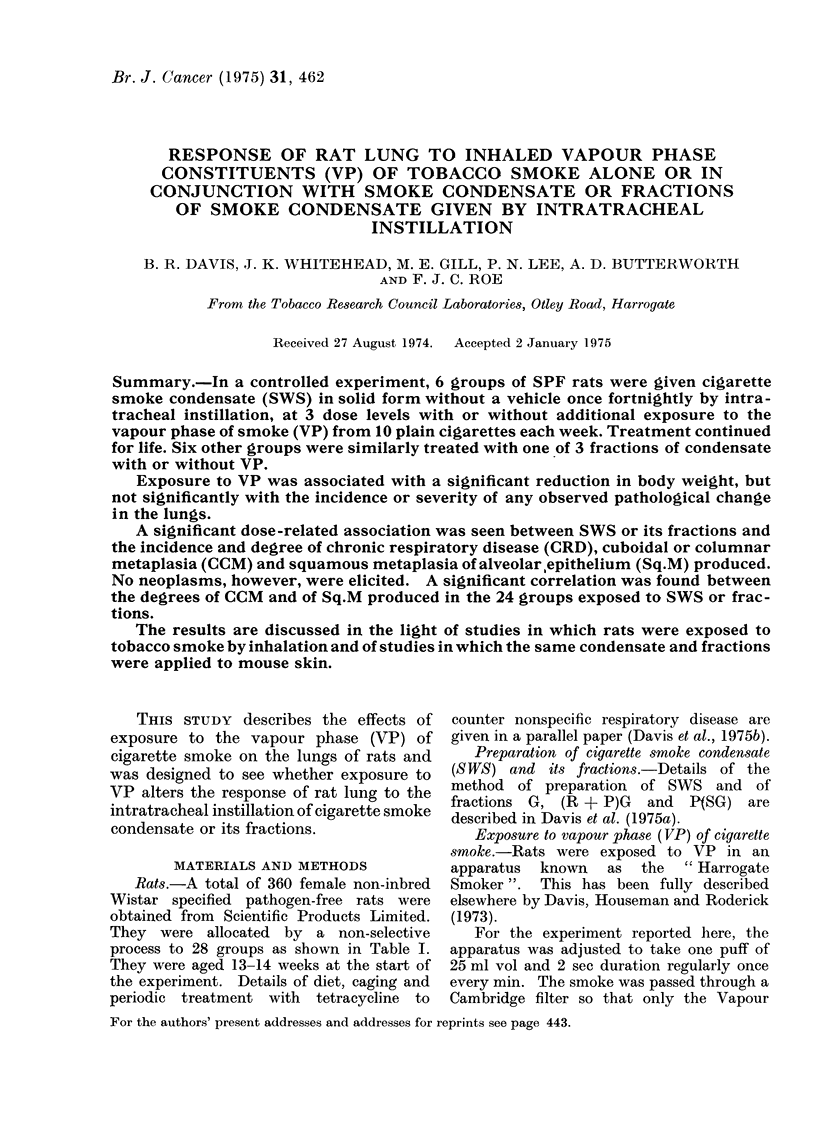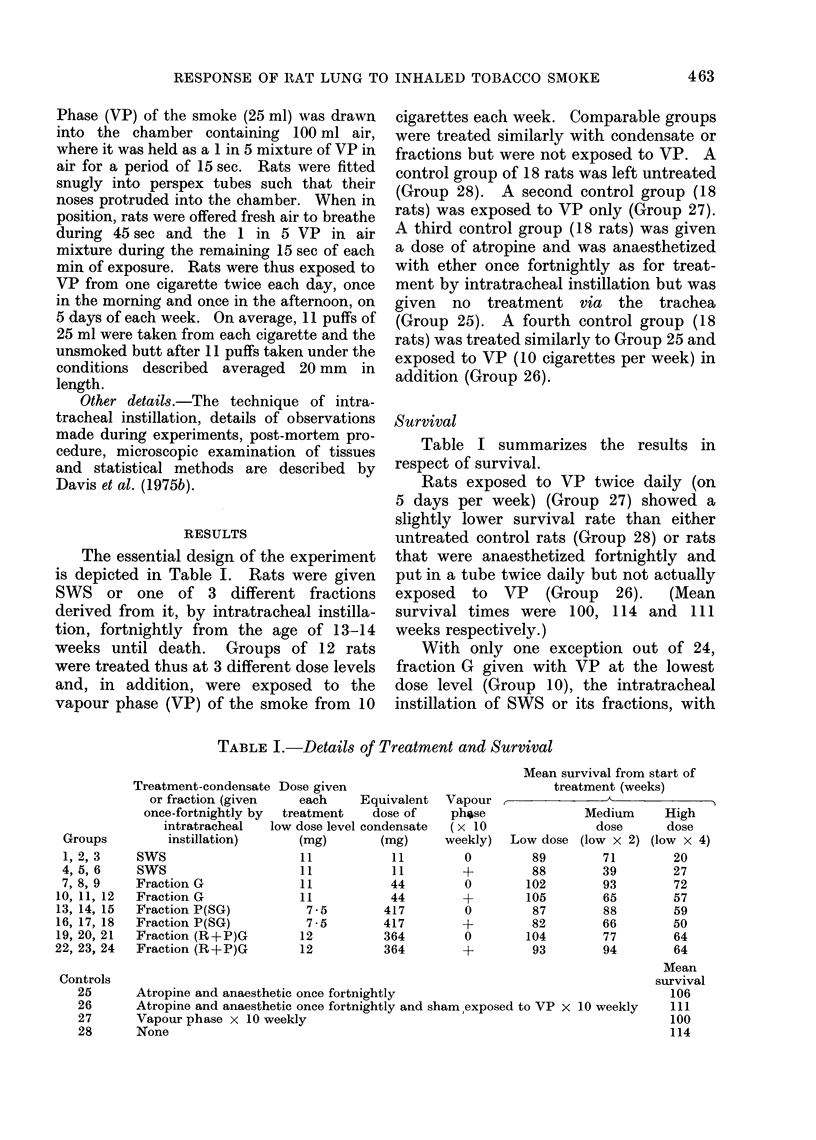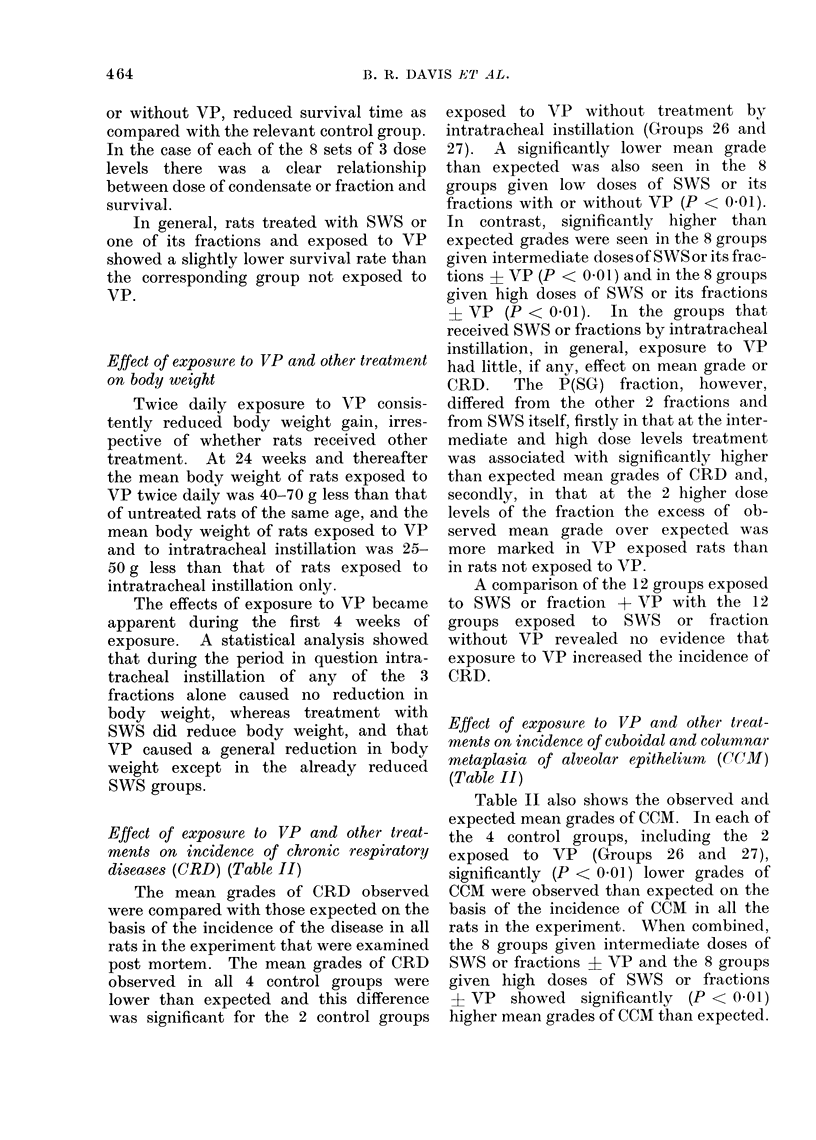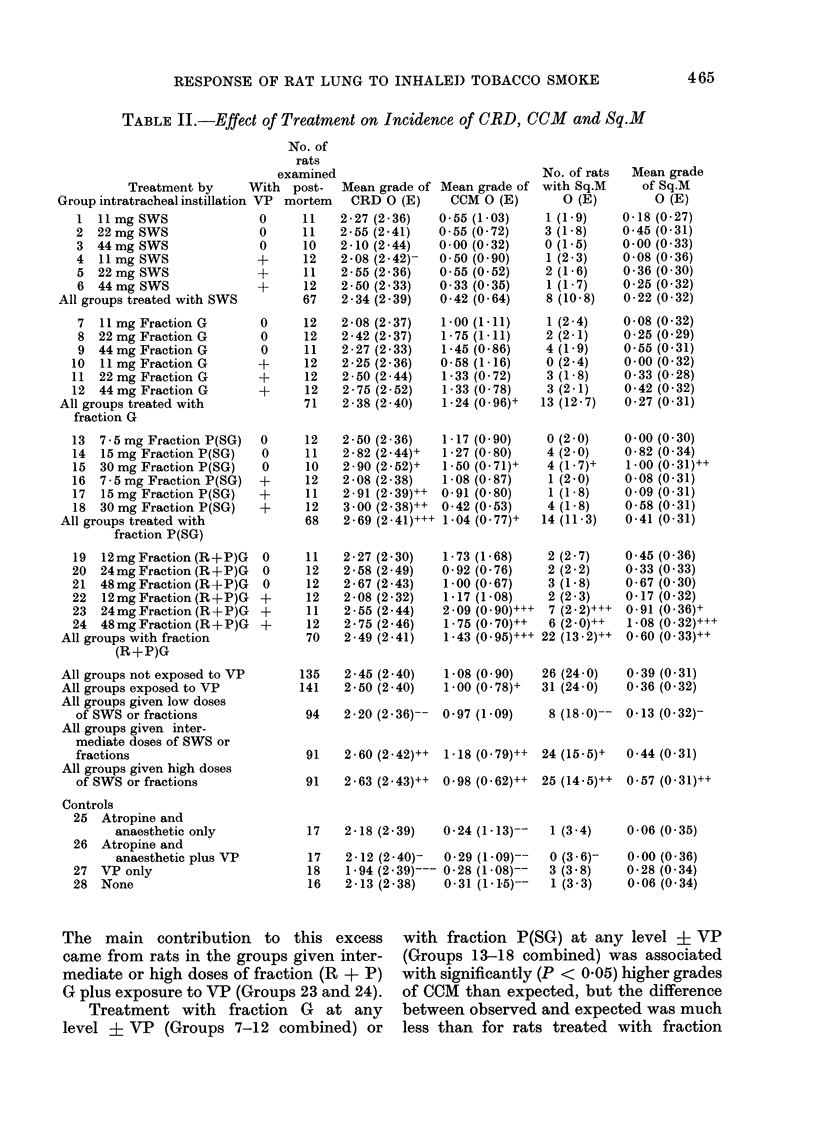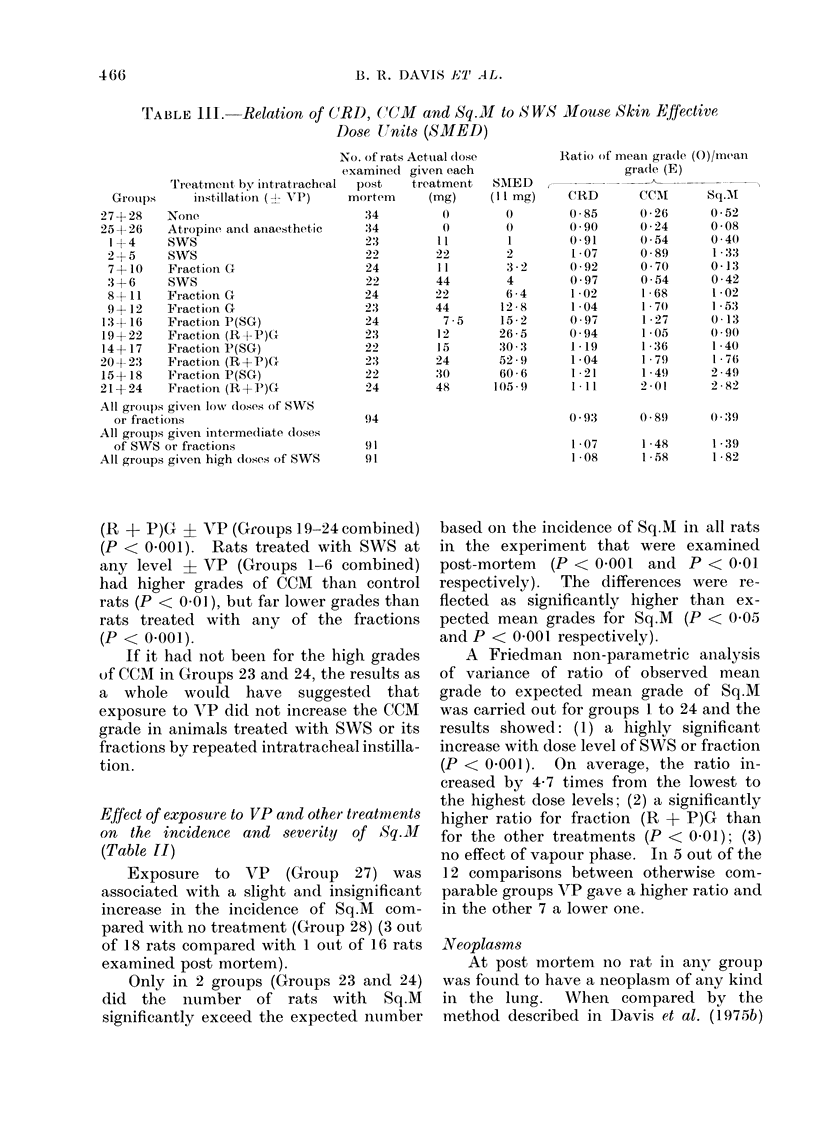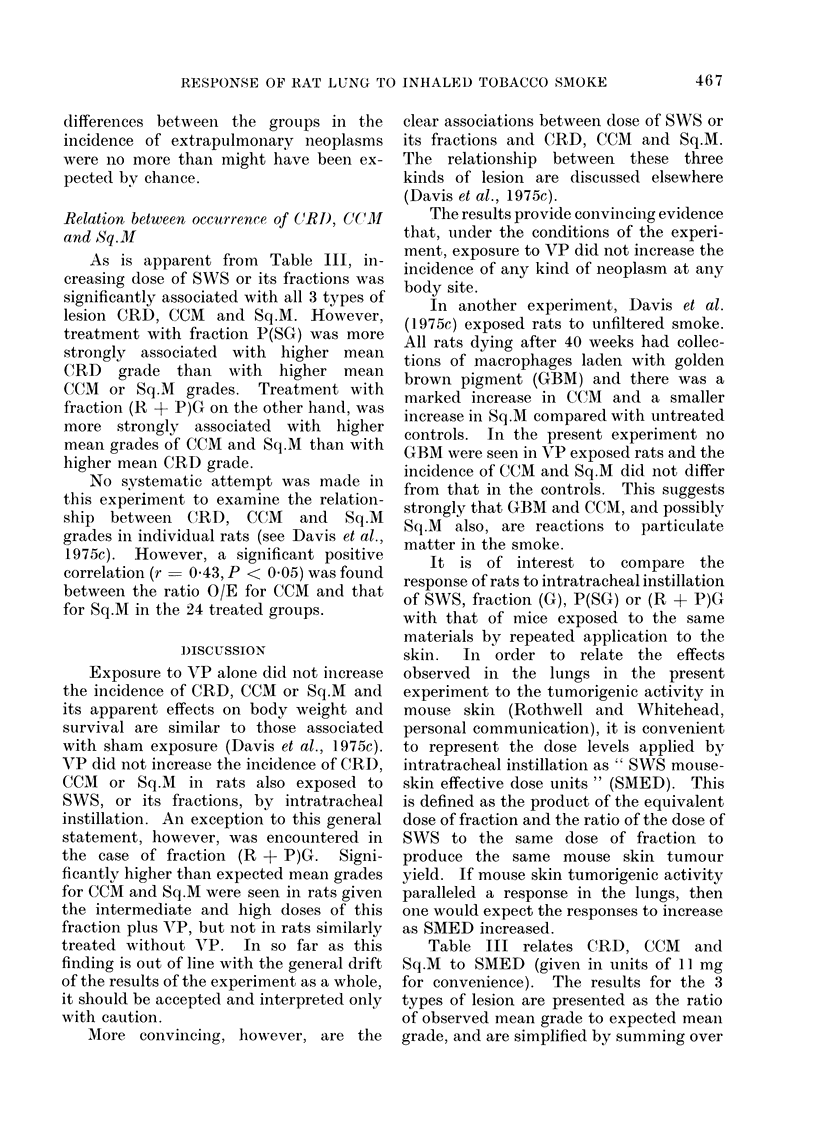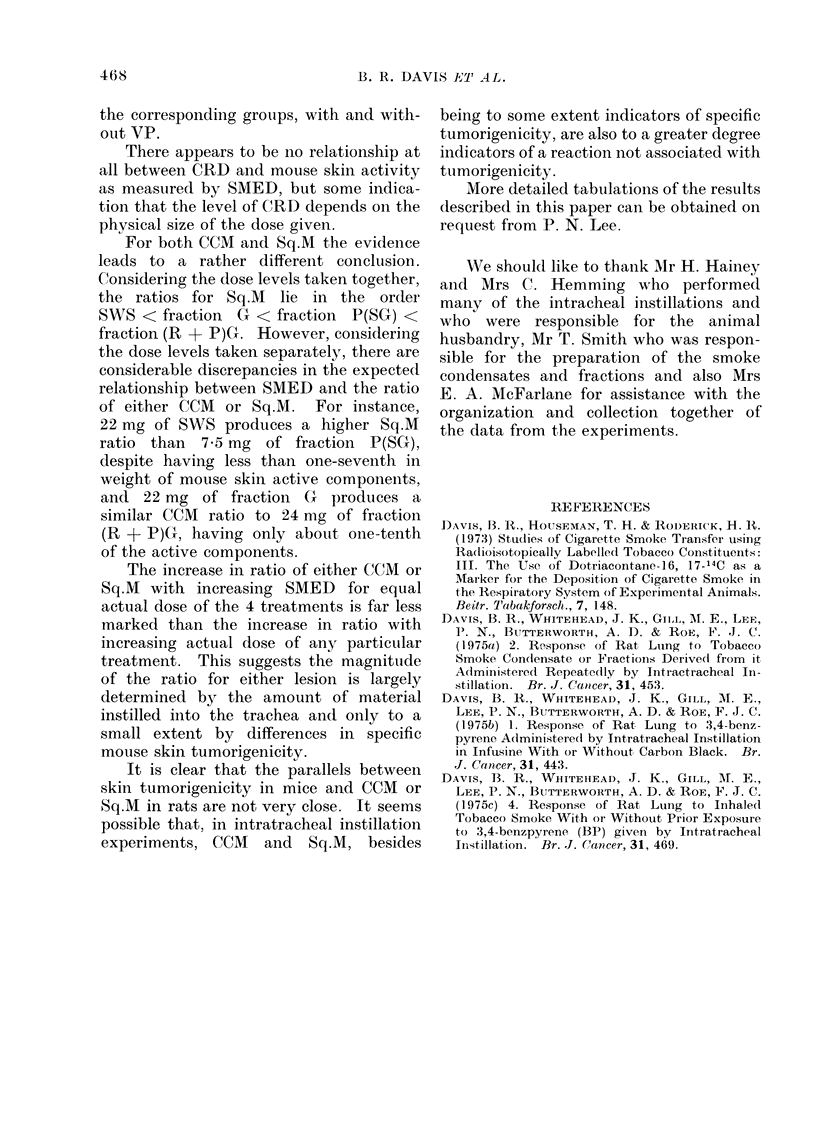# Response of rat lung to inhaled vapour phase constituents (VP) of tobacco smoke alone or in conjunction with smoke condensate or fractions of smoke condensate given by intratracheal instillation.

**DOI:** 10.1038/bjc.1975.86

**Published:** 1975-04

**Authors:** B. R. Davis, J. K. Whitehead, M. E. Gill, P. N. Lee, A. D. Butterworth, F. J. Roe

## Abstract

In a controlled experiment, 6 groups of SPF rats were given cigarette smoke condensate (SWS) in solid form without a vehicle once fortnightly by intratracheal instillation, at 3 dose levels with or without additional exposure to the vapour phase of smoke (VP) from 10 plain cigarettes each week. Treatment continued for life. Six other groups were similarly treated with one of 3 fractions of condensate with or without VP. Exposure to VP was associated with a significant reduction in body weight, but not signficantly with the incidence or severity of any observed pathological change in the lungs. A significant dose-related assoication was seen between SWS or its fractions and the incidence and degree of chronic respiratory disease (CRD), cuboidal or columnar metaplasia (CCM) and squamous metaplasia of alveolar epithelium (Sq.M) produced. No neoplasms, however, were elicited. A significant correlation was found between the degrees of CCM and of Sq.M produced in the 24 groups exposed to SWS or fractions. The results are discussed in the light of studies in which rats were exposed to tobacco smoke by inhalation and of studies in which the same condensate and fractions were applied to mouse skin.


*Br. J. Cancer* (1975) **31**, 462

### RESPONSE OF RAT LUNG TO INHALED VAPOUR PHASE CONSTITUENTS (VP) OF TOBACCO SMOKE ALONE OR IN CONJUNCTION WITH SMOKE CONDENSATE OR FRACTIONS OF SMOKE CONDENSATE GIVEN BY INTRATRACHEAL INSTILLATION

B. R. DAVIS, J. K. WHITEHEAD, M. E. GILL, P. N. LEE, A. D. BUTTERWORTH AND F. J. C. ROE

*From the Tobacco Research Council Laboratories, Otley Road, Harrogate*

Received 27 August 1974. Accepted 2 January 1975

**Summary.—In a controlled experiment, 6 groups of SPF rats were given cigarette smoke condensate (SWS) in solid form without a vehicle once fortnightly by intratracheal instillation, at 3 dose levels with or without additional exposure to the vapour phase of smoke (VP) from 10 plain cigarettes each week. Treatment continued for life. Six other groups were similarly treated with one of 3 fractions of condensate with or without VP.**

**Exposure to VP was associated with a significant reduction in body weight, but not significantly with the incidence or severity of any observed pathological change in the lungs.**

**A significant dose-related association was seen between SWS or its fractions and the incidence and degree of chronic respiratory disease (CRD), cuboidal or columnar metaplasia (CCM) and squamous metaplasia of alveolar epithelium (Sq.M) produced. No neoplasms, however, were elicited. A significant correlation was found between the degrees of CCM and of Sq.M produced in the 24 groups exposed to SWS or fractions.**

**The results are discussed in the light of studies in which rats were exposed to tobacco smoke by inhalation and of studies in which the same condensate and fractions were applied to mouse skin.**

THIS STUDY describes the effects of exposure to the vapour phase (VP) of cigarette smoke on the lungs of rats and was designed to see whether exposure to VP alters the response of rat lung to the intratracheal instillation of cigarette smoke condensate or its fractions.

### MATERIALS AND METHODS

*Rats.*—A total of 360 female non-inbred Wistar specified pathogen-free rats were obtained from Scientific Products Limited. They were allocated by a non-selective process to 28 groups as shown in Table I. They were aged 13–14 weeks at the start of the experiment. Details of diet, caging and periodic treatment with tetracycline to counter nonspecific respiratory disease are given in a parallel paper (Davis *et al.*, 1975*b*).

*Preparation of cigarette smoke condensate (SWS) and its fractions.*—Details of the method of preparation of SWS and of fractions G, (R + P)G and P(SG) are described in Davis *et al.* (1975*a*).

*Exposure to vapour phase (VP) of cigarette smoke.*—Rats were exposed to VP in an apparatus known as the "Harrogate Smoker". This has been fully described elsewhere by Davis, Houseman and Roderick (1973).

For the experiment reported here, the apparatus was adjusted to take one puff of 25 ml vol and 2 sec duration regularly once every min. The smoke was passed through a Cambridge filter so that only the Vapour

For the authors' present addresses and addresses for reprints see page 443.

Phase (VP) of the smoke (25 ml) was drawn into the chamber containing 100 ml air, where it was held as a 1 in 5 mixture of VP in air for a period of 15 sec. Rats were fitted snugly into perspex tubes such that their noses protruded into the chamber. When in position, rats were offered fresh air to breathe during 45 sec and the 1 in 5 VP in air mixture during the remaining 15 sec of each min of exposure. Rats were thus exposed to VP from one cigarette twice each day, once in the morning and once in the afternoon, on 5 days of each week. On average, 11 puffs of 25 ml were taken from each cigarette and the unsmoked butt after 11 puffs taken under the conditions described averaged 20 mm in length.

*Other details.*—The technique of intratracheal instillation, details of observations made during experiments, post-mortem procedure, microscopic examination of tissues and statistical methods are described by Davis *et al.* (1975*b*).

### RESULTS

The essential design of the experiment is depicted in Table I. Rats were given SWS or one of 3 different fractions derived from it, by intratracheal instillation, fortnightly from the age of 13–14 weeks until death. Groups of 12 rats were treated thus at 3 different dose levels and, in addition, were exposed to the vapour phase (VP) of the smoke from 10 cigarettes each week. Comparable groups were treated similarly with condensate or fractions but were not exposed to VP. A control group of 18 rats was left untreated (Group 28). A second control group (18 rats) was exposed to VP only (Group 27). A third control group (18 rats) was given a dose of atropine and was anaesthetized with ether once fortnightly as for treatment by intratracheal instillation but was given no treatment *via* the trachea (Group 25). A fourth control group (18 rats) was treated similarly to Group 25 and exposed to VP (10 cigarettes per week) in addition (Group 26).

### *Survival*

Table I summarizes the results in respect of survival.

Rats exposed to VP twice daily (on 5 days per week) (Group 27) showed a slightly lower survival rate than either untreated control rats (Group 28) or rats that were anaesthetized fortnightly and put in a tube twice daily but not actually exposed to VP (Group 26). (Mean survival times were 100, 114 and 111 weeks respectively.)

With only one exception out of 24, fraction G given with VP at the lowest dose level (Group 10), the intratracheal instillation of SWS or its fractions, with

TABLE I.—*Details of Treatment and Survival*

| Groups | Treatment-condensate or fraction (given once-fortnightly by intratracheal instillation) | Dose given each treatment low dose level (mg) | Equivalent dose of condensate (mg) | Vapour phase (× 10 weekly) | Mean survival from start of treatment (weeks) Low dose | Medium dose (low × 2) | High dose (low × 4) |
|---|---|---|---|---|---|---|---|
| 1, 2, 3 | SWS | 11 | 11 | 0 | 89 | 71 | 20 |
| 4, 5, 6 | SWS | 11 | 11 | + | 88 | 39 | 27 |
| 7, 8, 9 | Fraction G | 11 | 44 | 0 | 102 | 93 | 72 |
| 10, 11, 12 | Fraction G | 11 | 44 | + | 105 | 65 | 57 |
| 13, 14, 15 | Fraction P(SG) | 7·5 | 417 | 0 | 87 | 88 | 59 |
| 16, 17, 18 | Fraction P(SG) | 7·5 | 417 | + | 82 | 66 | 50 |
| 19, 20, 21 | Fraction (R+P)G | 12 | 364 | 0 | 104 | 77 | 64 |
| 22, 23, 24 | Fraction (R+P)G | 12 | 364 | + | 93 | 94 | 64 |

| Controls | | Mean survival |
|---|---|---|
| 25 | Atropine and anaesthetic once fortnightly | 106 |
| 26 | Atropine and anaesthetic once fortnightly and sham exposed to VP × 10 weekly | 111 |
| 27 | Vapour phase × 10 weekly | 100 |
| 28 | None | 114 |

or without VP, reduced survival time as compared with the relevant control group. In the case of each of the 8 sets of 3 dose levels there was a clear relationship between dose of condensate or fraction and survival.

In general, rats treated with SWS or one of its fractions and exposed to VP showed a slightly lower survival rate than the corresponding group not exposed to VP.

*Effect of exposure to VP and other treatment on body weight*

Twice daily exposure to VP consistently reduced body weight gain, irrespective of whether rats received other treatment. At 24 weeks and thereafter the mean body weight of rats exposed to VP twice daily was 40–70 g less than that of untreated rats of the same age, and the mean body weight of rats exposed to VP and to intratracheal instillation was 25–50 g less than that of rats exposed to intratracheal instillation only.

The effects of exposure to VP became apparent during the first 4 weeks of exposure. A statistical analysis showed that during the period in question intratracheal instillation of any of the 3 fractions alone caused no reduction in body weight, whereas treatment with SWS did reduce body weight, and that VP caused a general reduction in body weight except in the already reduced SWS groups.

*Effect of exposure to VP and other treatments on incidence of chronic respiratory diseases (CRD) (Table II)*

The mean grades of CRD observed were compared with those expected on the basis of the incidence of the disease in all rats in the experiment that were examined post mortem. The mean grades of CRD observed in all 4 control groups were lower than expected and this difference was significant for the 2 control groups exposed to VP without treatment by intratracheal instillation (Groups 26 and 27). A significantly lower mean grade than expected was also seen in the 8 groups given low doses of SWS or its fractions with or without VP ($P < 0{\cdot}01$). In contrast, significantly higher than expected grades were seen in the 8 groups given intermediate doses of SWS or its fractions $\pm$ VP ($P < 0{\cdot}01$) and in the 8 groups given high doses of SWS or its fractions $\pm$ VP ($P < 0{\cdot}01$). In the groups that received SWS or fractions by intratracheal instillation, in general, exposure to VP had little, if any, effect on mean grade or CRD. The P(SG) fraction, however, differed from the other 2 fractions and from SWS itself, firstly in that at the intermediate and high dose levels treatment was associated with significantly higher than expected mean grades of CRD and, secondly, in that at the 2 higher dose levels of the fraction the excess of observed mean grade over expected was more marked in VP exposed rats than in rats not exposed to VP.

A comparison of the 12 groups exposed to SWS or fraction + VP with the 12 groups exposed to SWS or fraction without VP revealed no evidence that exposure to VP increased the incidence of CRD.

*Effect of exposure to VP and other treatments on incidence of cuboidal and columnar metaplasia of alveolar epithelium (CCM) (Table II)*

Table II also shows the observed and expected mean grades of CCM. In each of the 4 control groups, including the 2 exposed to VP (Groups 26 and 27), significantly ($P < 0{\cdot}01$) lower grades of CCM were observed than expected on the basis of the incidence of CCM in all the rats in the experiment. When combined, the 8 groups given intermediate doses of SWS or fractions $\pm$ VP and the 8 groups given high doses of SWS or fractions $\pm$ VP showed significantly ($P < 0{\cdot}01$) higher mean grades of CCM than expected.

TABLE II.—*Effect of Treatment on Incidence of CRD, CCM and Sq.M*

| Group | Treatment by intratracheal instillation | With VP | No. of rats examined post-mortem | Mean grade of CRD O (E) | Mean grade of CCM O (E) | No. of rats with Sq.M O (E) | Mean grade of Sq.M O (E) |
|---|---|---|---|---|---|---|---|
| 1 | 11 mg SWS | 0 | 11 | 2·27 (2·36) | 0·55 (1·03) | 1 (1·9) | 0·18 (0·27) |
| 2 | 22 mg SWS | 0 | 11 | 2·55 (2·41) | 0·55 (0·72) | 3 (1·8) | 0·45 (0·31) |
| 3 | 44 mg SWS | 0 | 10 | 2·10 (2·44) | 0·00 (0·32) | 0 (1·5) | 0·00 (0·33) |
| 4 | 11 mg SWS | + | 12 | 2·08 (2·42)$^{-}$ | 0·50 (0·90) | 1 (2·3) | 0·08 (0·36) |
| 5 | 22 mg SWS | + | 11 | 2·55 (2·36) | 0·55 (0·52) | 2 (1·6) | 0·36 (0·30) |
| 6 | 44 mg SWS | + | 12 | 2·50 (2·33) | 0·33 (0·35) | 1 (1·7) | 0·25 (0·32) |
| All groups treated with SWS | | | 67 | 2·34 (2·39) | 0·42 (0·64) | 8 (10·8) | 0·22 (0·32) |
| 7 | 11 mg Fraction G | 0 | 12 | 2·08 (2·37) | 1·00 (1·11) | 1 (2·4) | 0·08 (0·32) |
| 8 | 22 mg Fraction G | 0 | 12 | 2·42 (2·37) | 1·75 (1·11) | 2 (2·1) | 0·25 (0·29) |
| 9 | 44 mg Fraction G | 0 | 11 | 2·27 (2·33) | 1·45 (0·86) | 4 (1·9) | 0·55 (0·31) |
| 10 | 11 mg Fraction G | + | 12 | 2·25 (2·36) | 0·58 (1·16) | 0 (2·4) | 0·00 (0·32) |
| 11 | 22 mg Fraction G | + | 12 | 2·50 (2·44) | 1·33 (0·72) | 3 (1·8) | 0·33 (0·28) |
| 12 | 44 mg Fraction G | + | 12 | 2·75 (2·52) | 1·33 (0·78) | 3 (2·1) | 0·42 (0·32) |
| All groups treated with fraction G | | | 71 | 2·38 (2·40) | 1·24 (0·96)$^{+}$ | 13 (12·7) | 0·27 (0·31) |
| 13 | 7·5 mg Fraction P(SG) | 0 | 12 | 2·50 (2·36) | 1·17 (0·90) | 0 (2·0) | 0·00 (0·30) |
| 14 | 15 mg Fraction P(SG) | 0 | 11 | 2·82 (2·44)$^{+}$ | 1·27 (0·80) | 4 (2·0) | 0·82 (0·34) |
| 15 | 30 mg Fraction P(SG) | 0 | 10 | 2·90 (2·52)$^{+}$ | 1·50 (0·71)$^{+}$ | 4 (1·7)$^{+}$ | 1·00 (0·31)$^{++}$ |
| 16 | 7·5 mg Fraction P(SG) | + | 12 | 2·08 (2·38) | 1·08 (0·87) | 1 (2·0) | 0·08 (0·31) |
| 17 | 15 mg Fraction P(SG) | + | 11 | 2·91 (2·39)$^{++}$ | 0·91 (0·80) | 1 (1·8) | 0·09 (0·31) |
| 18 | 30 mg Fraction P(SG) | + | 12 | 3·00 (2·38)$^{++}$ | 0·42 (0·53) | 4 (1·8) | 0·58 (0·31) |
| All groups treated with fraction P(SG) | | | 68 | 2·69 (2·41)$^{+++}$ | 1·04 (0·77)$^{+}$ | 14 (11·3) | 0·41 (0·31) |
| 19 | 12 mg Fraction (R+P)G | 0 | 11 | 2·27 (2·30) | 1·73 (1·68) | 2 (2·7) | 0·45 (0·36) |
| 20 | 24 mg Fraction (R+P)G | 0 | 12 | 2·58 (2·49) | 0·92 (0·76) | 2 (2·2) | 0·33 (0·33) |
| 21 | 48 mg Fraction (R+P)G | 0 | 12 | 2·67 (2·43) | 1·00 (0·67) | 3 (1·8) | 0·67 (0·30) |
| 22 | 12 mg Fraction (R+P)G | + | 12 | 2·08 (2·32) | 1·17 (1·08) | 2 (2·3) | 0·17 (0·32) |
| 23 | 24 mg Fraction (R+P)G | + | 11 | 2·55 (2·44) | 2·09 (0·90)$^{+++}$ | 7 (2·2)$^{+++}$ | 0·91 (0·36)$^{+}$ |
| 24 | 48 mg Fraction (R+P)G | + | 12 | 2·75 (2·46) | 1·75 (0·70)$^{++}$ | 6 (2·0)$^{++}$ | 1·08 (0·32)$^{+++}$ |
| All groups with fraction (R+P)G | | | 70 | 2·49 (2·41) | 1·43 (0·95)$^{+++}$ | 22 (13·2)$^{++}$ | 0·60 (0·33)$^{++}$ |
| All groups not exposed to VP | | | 135 | 2·45 (2·40) | 1·08 (0·90) | 26 (24·0) | 0·39 (0·31) |
| All groups exposed to VP | | | 141 | 2·50 (2·40) | 1·00 (0·78)$^{+}$ | 31 (24·0) | 0·36 (0·32) |
| All groups given low doses of SWS or fractions | | | 94 | 2·20 (2·36)$^{--}$ | 0·97 (1·09) | 8 (18·0)$^{--}$ | 0·13 (0·32)$^{-}$ |
| All groups given intermediate doses of SWS or fractions | | | 91 | 2·60 (2·42)$^{++}$ | 1·18 (0·79)$^{++}$ | 24 (15·5)$^{+}$ | 0·44 (0·31) |
| All groups given high doses of SWS or fractions | | | 91 | 2·63 (2·43)$^{++}$ | 0·98 (0·62)$^{++}$ | 25 (14·5)$^{++}$ | 0·57 (0·31)$^{++}$ |
| Controls | | | | | | | |
| 25 | Atropine and anaesthetic only | | 17 | 2·18 (2·39) | 0·24 (1·13)$^{--}$ | 1 (3·4) | 0·06 (0·35) |
| 26 | Atropine and anaesthetic plus VP | | 17 | 2·12 (2·40)$^{-}$ | 0·29 (1·09)$^{--}$ | 0 (3·6)$^{-}$ | 0·00 (0·36) |
| 27 | VP only | | 18 | 1·94 (2·39)$^{---}$ | 0·28 (1·08)$^{--}$ | 3 (3·8) | 0·28 (0·34) |
| 28 | None | | 16 | 2·13 (2·38) | 0·31 (1·15)$^{--}$ | 1 (3·3) | 0·06 (0·34) |

The main contribution to this excess came from rats in the groups given intermediate or high doses of fraction (R + P) G plus exposure to VP (Groups 23 and 24).

Treatment with fraction G at any level ± VP (Groups 7–12 combined) or with fraction P(SG) at any level ± VP (Groups 13–18 combined) was associated with significantly ($P < 0{\cdot}05$) higher grades of CCM than expected, but the difference between observed and expected was much less than for rats treated with fraction

TABLE III.—*Relation of CRD, CCM and Sq.M to SWS Mouse Skin Effective Dose Units (SMED)*

| Groups | Treatment by intratracheal instillation (± VP) | No. of rats examined post mortem | Actual dose given each treatment (mg) | SMED (11 mg) | Ratio of mean grade (O)/mean grade (E) | | |
|---|---|---|---|---|---|---|---|
| | | | | | CRD | CCM | Sq.M |
| 27+28 | None | 34 | 0 | 0 | 0·85 | 0·26 | 0·52 |
| 25+26 | Atropine and anaesthetic | 34 | 0 | 0 | 0·90 | 0·24 | 0·08 |
| 1+4 | SWS | 23 | 11 | 1 | 0·91 | 0·54 | 0·40 |
| 2+5 | SWS | 22 | 22 | 2 | 1·07 | 0·89 | 1·33 |
| 7+10 | Fraction G | 24 | 11 | 3·2 | 0·92 | 0·70 | 0·13 |
| 3+6 | SWS | 22 | 44 | 4 | 0·97 | 0·54 | 0·42 |
| 8+11 | Fraction G | 24 | 22 | 6·4 | 1·02 | 1·68 | 1·02 |
| 9+12 | Fraction G | 23 | 44 | 12·8 | 1·04 | 1·70 | 1·53 |
| 13+16 | Fraction P(SG) | 24 | 7·5 | 15·2 | 0·97 | 1·27 | 0·13 |
| 19+22 | Fraction (R+P)G | 23 | 12 | 26·5 | 0·94 | 1·05 | 0·90 |
| 14+17 | Fraction P(SG) | 22 | 15 | 30·3 | 1·19 | 1·36 | 1·40 |
| 20+23 | Fraction (R+P)G | 23 | 24 | 52·9 | 1·04 | 1·79 | 1·76 |
| 15+18 | Fraction P(SG) | 22 | 30 | 60·6 | 1·21 | 1·49 | 2·49 |
| 21+24 | Fraction (R+P)G | 24 | 48 | 105·9 | 1·11 | 2·01 | 2·82 |
| All groups given low doses of SWS or fractions | | 94 | | | 0·93 | 0·89 | 0·39 |
| All groups given intermediate doses of SWS or fractions | | 91 | | | 1·07 | 1·48 | 1·39 |
| All groups given high doses of SWS | | 91 | | | 1·08 | 1·58 | 1·82 |

(R + P)G ± VP (Groups 19–24 combined) ($P < 0{\cdot}001$). Rats treated with SWS at any level ± VP (Groups 1–6 combined) had higher grades of CCM than control rats ($P < 0{\cdot}01$), but far lower grades than rats treated with any of the fractions ($P < 0{\cdot}001$).

If it had not been for the high grades of CCM in Groups 23 and 24, the results as a whole would have suggested that exposure to VP did not increase the CCM grade in animals treated with SWS or its fractions by repeated intratracheal instillation.

*Effect of exposure to VP and other treatments on the incidence and severity of Sq.M (Table II)*

Exposure to VP (Group 27) was associated with a slight and insignificant increase in the incidence of Sq.M compared with no treatment (Group 28) (3 out of 18 rats compared with 1 out of 16 rats examined post mortem).

Only in 2 groups (Groups 23 and 24) did the number of rats with Sq.M significantly exceed the expected number based on the incidence of Sq.M in all rats in the experiment that were examined post-mortem ($P < 0{\cdot}001$ and $P < 0{\cdot}01$ respectively). The differences were reflected as significantly higher than expected mean grades for Sq.M ($P < 0{\cdot}05$ and $P < 0{\cdot}001$ respectively).

A Friedman non-parametric analysis of variance of ratio of observed mean grade to expected mean grade of Sq.M was carried out for groups 1 to 24 and the results showed: (1) a highly significant increase with dose level of SWS or fraction ($P < 0{\cdot}001$). On average, the ratio increased by 4·7 times from the lowest to the highest dose levels; (2) a significantly higher ratio for fraction (R + P)G than for the other treatments ($P < 0{\cdot}01$); (3) no effect of vapour phase. In 5 out of the 12 comparisons between otherwise comparable groups VP gave a higher ratio and in the other 7 a lower one.

*Neoplasms*

At post mortem no rat in any group was found to have a neoplasm of any kind in the lung. When compared by the method described in Davis *et al.* (1975*b*)

differences between the groups in the incidence of extrapulmonary neoplasms were no more than might have been expected by chance.

*Relation between occurrence of CRD, CCM and Sq.M*

As is apparent from Table III, increasing dose of SWS or its fractions was significantly associated with all 3 types of lesion CRD, CCM and Sq.M. However, treatment with fraction P(SG) was more strongly associated with higher mean CRD grade than with higher mean CCM or Sq.M grades. Treatment with fraction (R + P)G on the other hand, was more strongly associated with higher mean grades of CCM and Sq.M than with higher mean CRD grade.

No systematic attempt was made in this experiment to examine the relationship between CRD, CCM and Sq.M grades in individual rats (see Davis *et al.*, 1975*c*). However, a significant positive correlation ($r = 0{\cdot}43, P < 0{\cdot}05$) was found between the ratio O/E for CCM and that for Sq.M in the 24 treated groups.

DISCUSSION

Exposure to VP alone did not increase the incidence of CRD, CCM or Sq.M and its apparent effects on body weight and survival are similar to those associated with sham exposure (Davis *et al.*, 1975*c*). VP did not increase the incidence of CRD, CCM or Sq.M in rats also exposed to SWS, or its fractions, by intratracheal instillation. An exception to this general statement, however, was encountered in the case of fraction (R + P)G. Significantly higher than expected mean grades for CCM and Sq.M were seen in rats given the intermediate and high doses of this fraction plus VP, but not in rats similarly treated without VP. In so far as this finding is out of line with the general drift of the results of the experiment as a whole, it should be accepted and interpreted only with caution.

More convincing, however, are the clear associations between dose of SWS or its fractions and CRD, CCM and Sq.M. The relationship between these three kinds of lesion are discussed elsewhere (Davis *et al.*, 1975*c*).

The results provide convincing evidence that, under the conditions of the experiment, exposure to VP did not increase the incidence of any kind of neoplasm at any body site.

In another experiment, Davis *et al.* (1975*c*) exposed rats to unfiltered smoke. All rats dying after 40 weeks had collections of macrophages laden with golden brown pigment (GBM) and there was a marked increase in CCM and a smaller increase in Sq.M compared with untreated controls. In the present experiment no GBM were seen in VP exposed rats and the incidence of CCM and Sq.M did not differ from that in the controls. This suggests strongly that GBM and CCM, and possibly Sq.M also, are reactions to particulate matter in the smoke.

It is of interest to compare the response of rats to intratracheal instillation of SWS, fraction (G), P(SG) or (R + P)G with that of mice exposed to the same materials by repeated application to the skin. In order to relate the effects observed in the lungs in the present experiment to the tumorigenic activity in mouse skin (Rothwell and Whitehead, personal communication), it is convenient to represent the dose levels applied by intratracheal instillation as " SWS mouse-skin effective dose units " (SMED). This is defined as the product of the equivalent dose of fraction and the ratio of the dose of SWS to the same dose of fraction to produce the same mouse skin tumour yield. If mouse skin tumorigenic activity paralleled a response in the lungs, then one would expect the responses to increase as SMED increased.

Table III relates CRD, CCM and Sq.M to SMED (given in units of 11 mg for convenience). The results for the 3 types of lesion are presented as the ratio of observed mean grade to expected mean grade, and are simplified by summing over the corresponding groups, with and without VP.

There appears to be no relationship at all between CRD and mouse skin activity as measured by SMED, but some indication that the level of CRD depends on the physical size of the dose given.

For both CCM and Sq.M the evidence leads to a rather different conclusion. Considering the dose levels taken together, the ratios for Sq.M lie in the order SWS < fraction G < fraction P(SG) < fraction (R + P)G. However, considering the dose levels taken separately, there are considerable discrepancies in the expected relationship between SMED and the ratio of either CCM or Sq.M. For instance, 22 mg of SWS produces a higher Sq.M ratio than 7·5 mg of fraction P(SG), despite having less than one-seventh in weight of mouse skin active components, and 22 mg of fraction G produces a similar CCM ratio to 24 mg of fraction (R + P)G, having only about one-tenth of the active components.

The increase in ratio of either CCM or Sq.M with increasing SMED for equal actual dose of the 4 treatments is far less marked than the increase in ratio with increasing actual dose of any particular treatment. This suggests the magnitude of the ratio for either lesion is largely determined by the amount of material instilled into the trachea and only to a small extent by differences in specific mouse skin tumorigenicity.

It is clear that the parallels between skin tumorigenicity in mice and CCM or Sq.M in rats are not very close. It seems possible that, in intratracheal instillation experiments, CCM and Sq.M, besides being to some extent indicators of specific tumorigenicity, are also to a greater degree indicators of a reaction not associated with tumorigenicity.

More detailed tabulations of the results described in this paper can be obtained on request from P. N. Lee.

We should like to thank Mr H. Hainey and Mrs C. Hemming who performed many of the intracheal instillations and who were responsible for the animal husbandry, Mr T. Smith who was responsible for the preparation of the smoke condensates and fractions and also Mrs E. A. McFarlane for assistance with the organization and collection together of the data from the experiments.

### REFERENCES

DAVIS, B. R., HOUSEMAN, T. H. & RODERICK, H. R. (1973) Studies of Cigarette Smoke Transfer using Radioisotopically Labelled Tobacco Constituents: III. The Use of Dotriacontane-16, 17-$^{14}$C as a Marker for the Deposition of Cigarette Smoke in the Respiratory System of Experimental Animals. *Beitr. Tabakforsch.*, **7**, 148.

DAVIS, B. R., WHITEHEAD, J. K., GILL, M. E., LEE, P. N., BUTTERWORTH, A. D. & ROE, F. J. C. (1975*a*) 2. Response of Rat Lung to Tobacco Smoke Condensate or Fractions Derived from it Administered Repeatedly by Intractracheal Instillation. *Br. J. Cancer*, **31**, 453.

DAVIS, B. R., WHITEHEAD, J. K., GILL, M. E., LEE, P. N., BUTTERWORTH, A. D. & ROE, F. J. C. (1975*b*) 1. Response of Rat Lung to 3,4-benzpyrene Administered by Intratracheal Instillation in Infusine With or Without Carbon Black. *Br. J. Cancer*, **31**, 443.

DAVIS, B. R., WHITEHEAD, J. K., GILL, M. E., LEE, P. N., BUTTERWORTH, A. D. & ROE, F. J. C. (1975*c*) 4. Response of Rat Lung to Inhaled Tobacco Smoke With or Without Prior Exposure to 3,4-benzpyrene (BP) given by Intratracheal Instillation. *Br. J. Cancer*, **31**, 469.